# Supplementary material for: Risk Assessment and Sources Apportionment of Toxic Metals in Two Commonly Consumed Fishes from a Subtropical Estuarine Wetland System
Source: Biology (Basel). 2024 Apr 14;13(4):260. doi: 10.3390/biology13040260 (PMC11047917; doi:10.3390/biology13040260)
Supplement: Supplementary file 1 [file biology-13-00260-s001.zip › biology-2910150-supplementary.pdf]

Supplementary tables

Risk Assessment and Sources Apportionment of Toxic Metals in Two Commonly Consumed Fishes from a Subtropical Estuarine Wetland System

Table S1. MDLs (mg/kg) for EDXRF (Epsilon 5) and comparative values of the measured and certified concentrations of standard reference materials (Marine sediment, IAEA 433).

Elements	MDL (mg/kg)	Measured Conc. (mg/kg)		Certified Conc. (mg/L)	RE (%)	CV (%)
		Mean	SD			
Cr	0.89	76.96	0.33	77.2	0.32	0.43
Cu	0.24	30.63	0.34	32.7	6.33	1.11
Zn	0.32	72.66	0.87	74.8	2.87	1.20
Pb	0.55	22.45	0.08	22.3	1.54	0.38
